# Nonlinear Conductivity and Thermal Stability of Anti-Corona Epoxy Resin Nanocomposites

**DOI:** 10.3390/polym16091296

**Published:** 2024-05-05

**Authors:** Yanli Liu, Junguo Gao, Ning Guo, Jiaming Sun, Haitao Hu, Xiaohong Chi

**Affiliations:** 1 Key Laboratory of Engineering Dielectrics and Its Application, Ministry of Education, College of Electrical and Electronic Engineering, Harbin University of Science and Technology, Harbin 150080, China; liuyanli@hrbust.edu.cn (Y.L.); tad@hrbust.edu.cn (N.G.); sjm3852@163.com (J.S.); huhaitaodianqi@hrbust.edu.cn (H.H.); 2 State Key Laboratory of Electrical Insulation and Power Equipment, School of Electrical Engineering, Xi’an Jiaotong University, Xi’an 710000, China; hrbustcxh@xjtu.edu.cn

**Keywords:** epoxy resin, glass-transition temperature, nanodielectrics, thermal stability, nonlinear conductivity

## Abstract

The long-term operation of motors induces substantial alterations in the surface conductivity and nonlinear coefficient of anti-corona paint, diminishing its efficacy and jeopardizing the longevity of large motors. Hence, the development of high-performance anti-corona paint holds paramount importance in ensuring motor safety. In this study, we integrate two nano-fillers, namely silicon carbide (SiC) and organic montmorillonite (O-MMT), into a composite matrix comprising micron silicon carbide and epoxy resin (SiC/EP). Subsequently, three distinct types of anti-corona paint are formulated: SiC/EP, Nano-SiC/EP, and O-MMT/SiC/EP. Remarkably, O-MMT/SiC/EP exhibits a glass transition temperature about 25 °C higher than that of SiC/EP, underscoring its superior thermal properties. Moreover, the introduction of nano-fillers markedly augments the surface conductivity of the anti-corona paint. Aging tests, conducted across varying temperatures, unveil a notable reduction in the fluctuation range of surface conductivity post-aging. Initially, the nonlinear coefficients exhibit a declining trend, succeeded by an ascending trajectory. The O-MMT/SiC/EP composite displays a maximum nonlinearity coefficient of 1.465 and a minimum of 1.382. Furthermore, the incorporation of nanofillers amplifies the dielectric thermal stability of epoxy resin composites, with O-MMT/SiC/EP showcasing the pinnacle of thermal endurance. Overall, our findings elucidate the efficacy of nano-fillers in enhancing the performance and longevity of anti-corona paint, particularly highlighting the exceptional attributes of the O-MMT/SiC/EP composite in bolstering motor safety through improved thermal stability and electrical properties.

## 1. Introduction

Polymer materials play a crucial role in daily life, particularly polymer nanocomposites filled with nano-fillers, and their potential applications are immeasurable. These high-performance composites find applications not only in the fabrication of efficient batteries for mobile devices and electric vehicles [1,2,3], but also as lightweight and efficient materials for electromagnetic shielding in communications and aerospace equipment [4,5,6]. The exceptional performance of these composites in the application of large motors, coupled with their suitability as anti-corona materials for such motors, deserves special mention.

With the increase in voltage level, the electric field distortion at the stator bar end of a large motor will gradually intensify [7,8]. Severe electric field distortion can result in corona discharge of the motor. Extensive research has been conducted to optimize the electric field intensity at this critical interface [9,10]. Therefore, it is important to develop efficient anti-corona materials. The utilization of silicon carbide (SiC) is attributed to its exceptional attributes, including a high melting point, superior mechanical properties, excellent high-temperature stability, corrosion resistance, and more. Consequently, it finds extensive application in the manufacturing of large motors. The anti-corona paint, which incorporates silicon carbide as a filler, exhibits a distinct nonlinear characteristic where resistance substantially decreases with increased electric field intensity, notably improving the distribution of electric fields at the stator bar terminus [11,12,13]. However, prolonged operational periods induce significant variations in both resistance value and nonlinear coefficients of anti-corona paint due to aging, culminating in partial discharges at stator bar ends [14,15]. Therefore, it is imperative to develop long-lasting polymer composites with anti-corona properties capable of minimizing fluctuations in resistance values and nonlinear coefficients. The properties of nano-silicon carbide surpass those of traditional silicon carbide materials, thereby effectively enhancing the electrical and mechanical characteristics of polymer composites [16,17,18]. This offers a broader perspective for the research and development of anti-corona materials in large-scale motors.

Epoxy resins are thermosetting polymers, and epoxy resin adhesive possesses numerous advantages that other adhesives lack, making it widely utilized. The performance of a composite dielectric can be significantly influenced by the incorporation of nanoparticles into epoxy resin, as indicated by several scientific studies. Alumina nanofibers were successfully synthesized via the sol–gel template method by Pavel V. Krivoshapkin et al., and incorporating them into epoxy composites led to significant enhancements in both thermal stability and mechanical strength properties [19]. Pan Hu et al. successfully functionalized the surface of nano TiO2 by grafting a silane coupling agent, KH570, onto its hydroxyl groups, resulting in the preparation of an epoxy resin nanocomposite material. This modification significantly enhanced the impact strength of the substrate to 36.78 KJ/m2 [20]. Some scholars have also enhanced the fracture toughness of epoxy resins by incorporating various nano-fillers [21,22,23].

Epoxy resin is a crucial component in anti-corona paint, as it serves as an adhesive. The effectiveness of the dielectrics made from epoxy resin and silicon carbide composites has a direct impact on the dielectric properties and thermal stability of paint. Nano fillers can enhance the hardness, wear resistance, corrosion resistance, and other physical and chemical properties of epoxy resin, so as to improve the protective effect and service life of anti-corona paint. Chi et al., through the integration of silicon carbide/silicon dioxide core/shell structured fillers into epoxy resin (EP), observed enhancements in both nonlinear coefficients and breakdown field strengths of resulting composites. Additionally, the nonlinear characteristics of EP were further enhanced by the incorporation of the silicon carbide whisker (SiCw) [24,25]. Investigating the DC voltage characteristics of five epoxy-based composites, H. Hu et al. noted increased conductivity and nonlinear coefficients with increasing inorganic filler content, utilizing a single filler type [26]. Sun et al. investigated how different sizes of zinc oxide affected the electrical characteristics of MMT/SiC/EP micro-nano composites. They emphasized that tetra-needle-shaped zinc oxide promoted the formation of highly conductive pathways, leading to improved electrical conductivity and nonlinear coefficients [27,28]. While extensive research on epoxy-based composite dielectrics has laid the theoretical groundwork for superior anti-corona paint preparation, these studies primarily focus on nanofiller type, content, and dimensions, overlooking changes in the electrical properties and thermal stability of the composite dielectric post-prolonged motor operation. Over time, the polymers in anti-corona paint undergo thermal activation in an oxygen-rich environment and are prone to thermal oxidation aging, resulting in the continuous absorption of oxygen to produce hydroperoxides. The instability of hydroperoxides can lead to backbone rearrangement, chain breakage, or cross-linking, thereby diminishing the properties of polymer materials [29,30] and affecting the thermal stability of anti-corona paint.

The modification of an epoxy resin composite matrix by nanoparticles, and especially the change in its thermal stability, is relatively rare in the existing literature. In this paper, two types of epoxy resin micro-nano composite dielectric coatings were studied, and the changes in microstructure, surface conductivity, and non-linear coefficient after prolonged surface aging were investigated. The objective was to investigate the thermal stability of epoxy composites modified with nanoparticles by examining the glass transition temperature, surface conductivity change rate, and nonlinear coefficient variation under different aging durations. This study provides new insights into the corona protection of epoxy composites based on nanoparticles and establishes a theoretical foundation for developing highly thermally stable anti-corona coatings using micro-nano composite systems of epoxy resin, thereby advancing the progress and application of anti-corona paint technology.

## 2. Material Preparation and Test Methods

### 2.1. Raw Materials and Test Equipment

In experiments, the selection of appropriate materials and the adherence to correct procedures have a significant impact on results. Improper material selection or deviations in testing processes can lead to inaccurate or irreproducible outcomes. Therefore, a careful consideration of experimental materials and test process design is necessary prior to conducting experiments to ensure the reliability and accuracy of results. Additionally, a strict adherence to laboratory safety regulations during experimentation is crucial for maintaining safety and control over operations. Only through proper material selection and adherence to correct testing procedures can reliable experimental results be obtained, providing strong support for scientific research.

To prepare the objective composites used for anti-corona paint, Table 1 displays a list of necessary raw materials for conducting the test.

**Figure 1:**
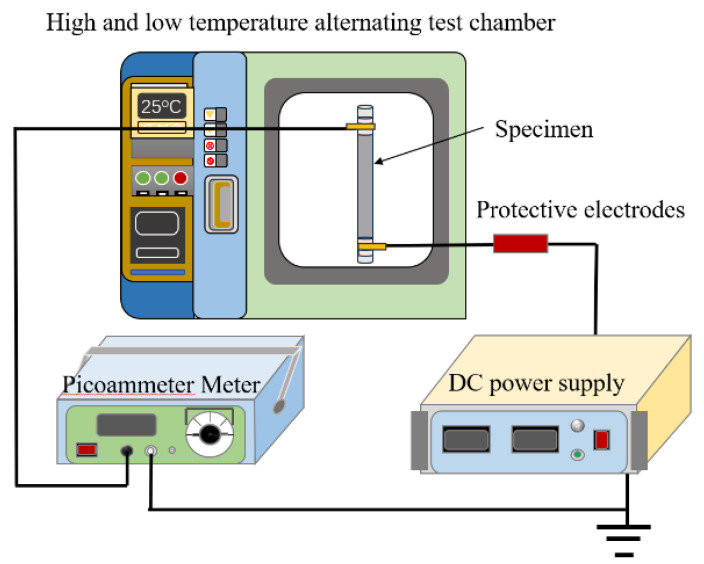
Schematic test system for the surface conductivity of anti-corona paints.

For measuring the surface electrical conductivity of the anti-corona paint specimen made of the benchmark and prepared composites, a self-constructed two-electrode test system was employed, as schematically depicted in Figure 1, consisting of the following experimental apparatus: a high-voltage DC regulated power supply (DW-P153-5ACF3, 0~15 kV, Dongwen High Voltage Power Supply Co., Ltd., Tianjin, China), the EST122 Picoammeter (10^−4^~10^−14^ A, Beijing Chuangtou Science and Technology Co., Ltd., Beijing, China), and a high–low temperature alternating test chamber (GP/GDW150, Shanghai Guangpin Test Equipment Manufacturing Co., Ltd., Shanghai, China).

### 2.2. Material Preparation

#### 2.2.1. Pretreatment of Inorganic Fillers

**Figure 2:**
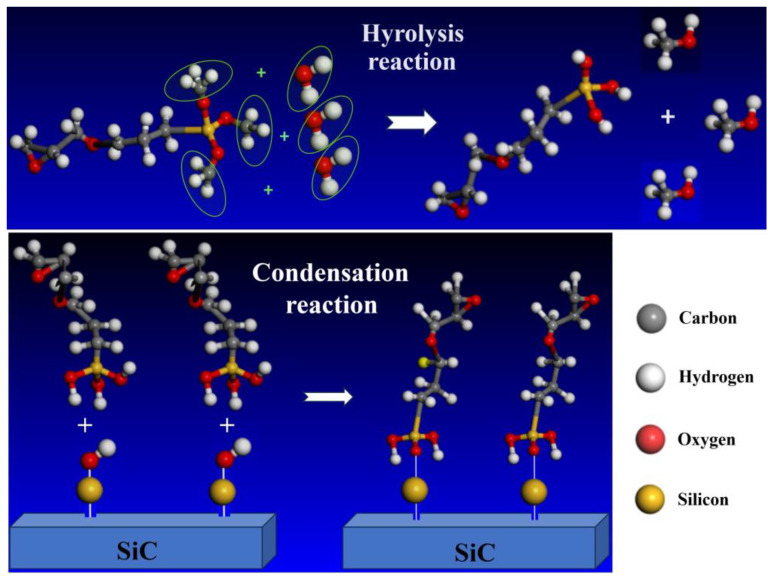
Schematic hydrolysis (**top panel**) and polycondensation (**bottom panel**) reactions of silane coupling agent.

To enhance the compatibility between the epoxy resin matrix and inorganic nanofillers, a pre-treatment involving organic chemical modifications was conducted on SiC and MMT nanoparticles prior to the preparation of their epoxy resin composites. The modification process and mechanism of nano silicon carbide are as follows. Firstly, the pristine SiC powder was dried in an oven. Then, the silane coupling agent KH560 with a mass fraction of 37.5% was introduced into acetone while maintaining constant agitation. Finally, the nm-SiC with a mass fraction of 25.0% was added to the acetone solution [31]. The prepared solution was subsequently transferred into a three-necked flask, followed by a soaking period of 2 h. After that, the solution was stirred at a speed of 1500 r/min and thoroughly mixed in a water bath maintained at 50 °C. Afterwards, the surface-modified silicon carbide nanoparticles (nano-SiC) were obtained through a complete sequence of treatments that include centrifugal separation, ultrasonic washing, standing, repeated washing, drying, grinding, and sieving. The molecular formula of a silane coupling agent is generally Y-(CH_2_) *n*-SiX_3_ (where *n* = 0–3, X represents hydrolysis groups, and Y represents organic functional groups). The hydrolysis of these groups will produce silanol (Si(OH)_3_). In other words, after the hydrolysis of the silane coupling agent, a hydroxyl group is produced. This allows the silane coupling agent to react with the silicon hydroxyl group on the surface of the nano-silicon carbide. As a result, one end of the silane coupling agent can be connected to the surface of nano-silicon carbide, while the other end can be connected to the organic matrix [32]. The chemical reaction mechanism is schematically shown in Figure 2.

**Figure 3:**
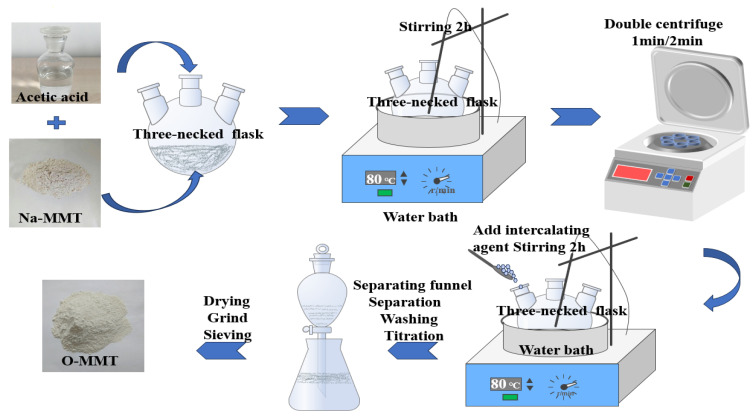
Flow chart of preparation of O-MMT nanoclusters.

The surface modification of the nano-scaled MMT material to finally achieve organic modified nano-MMT (O-MMT) was fulfilled by the chemical synthesis processes, as schematically shown in Figure 3. The preparation process of organic montmorillonite is as follows.

(1) To weigh the solid material, 20 g of Naki montmorillonite was taken into a three-necked flask, and 2.8 g of intercalation agent was taken into a beaker for later use.
(2) To prepare the acid solution, 500 mL of distilled water was poured into a beaker using a measuring cylinder, and then 0.5 mL of acetic acid solution was injected into the distilled water using a needle tube. After that, the mixture was stirred well with a glass rod. Next, 300 mL of the aforementioned acid solution was added to a three-necked flask containing montmorillonite and stirred to dislodge any montmorillonite stuck to the bottom of the flask.
(3) The flask was heated in a water bath at 80 °C while stirring for 2 h.
(4) The solution was stirred and then transferred into the centrifuge, where it underwent two rounds of centrifugation.
(5) Then, 50 mL of the slightly acidic solution obtained in step (2) was added to a beaker containing octadecyl trimethyl ammonium chloride, as described in (1). The solution was thoroughly stirred and then transferred, along with the remaining acid, into a three-necked flask. Subsequently, the mixture-containing three-necked flask was immersed in an 80 °C water bath and stirred continuously for a duration of 2 h.
(6) After being subjected to multiple cycles of standing, washing, filtering, drying, grinding, and sieving processes, the organically modified montmorillonite (O-MMT) was successfully obtained by putting the mixed solution into the separator funnel and allowing it to sit for 24 h.

#### 2.2.2. Preparation of Epoxy Resin Composites

**Figure 4:**
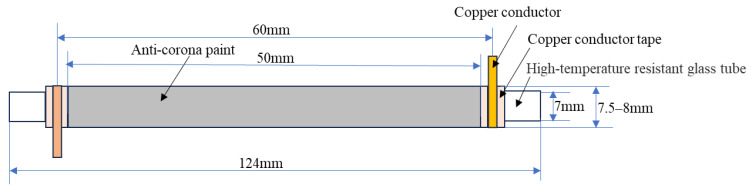
Anti-corona paint geometry of epoxy resin micron-nano composites.

The epoxy resin micro-nano composite anti-corona paint was prepared using the water bath blending method, as follows:

(1) The inorganic filler was pretreated, and then the modified nanoparticles were cleaned using low mineralization water with a pH of 7. After sufficient cleaning, the nanoparticles were vacuum-dried to remove any remaining water.
(2) The composite matrix SiC/EP was mixed with nanoparticles of SiC and MMT fillers, respectively, both modified with a mass fraction of 1 wt%. This choice of mass fraction (1 wt%) for the two nano-fillers aims to prevent agglomeration in the prepared composite materials. Furthermore, it ensured that the resulting composite materials exhibited good dielectric properties, as evidenced by our previously published article [33]. Mechanical stirring was performed on these two mixtures at room temperature for 2 h at a speed of 1500 r/min.
(3) The curing agent 593 was added to each mixture in sequence. The amount of curing agent 593 added was equal to 10% of the mass of each composite matrix. The main characteristics of the curing agent 593 include fast cure speed, high temperature resistance, and good chemical corrosion resistance. The resulting mixture was then mechanically stirred at room temperature for a duration of 30 min to ultimately obtain the anti-corona paint mixture.
(4) The outer wall of the high-temperature resistant glass tube was evenly coated with a composite material mixture, and left to cure for 24 h at room temperature, resulting in the formation of the preform for the glass tube.
(5) The glass tube prefabricated sample coating was equipped with copper conductive tape on both sides. A copper wire was wound around the copper conductive tape, serving as an electrode for convenient testing purposes. Figure 4 illustrates the final samples to be tested, which were coated with SiC/EP, Nano-SiC/EP, and O-MMT/SiC/EP anti-corona paint.

### 2.3. Material Characterization

The chemical components of O-MMT material were characterized by infrared spectroscopy, as implemented by a Fourier transform infrared (FTIR) spectrometer (Nicolet iS5, Co. Ltd., Thermo Fisher Scientific Co., Ltd., Waltham, MA, USA). The test samples were prepared using the KBr tablet method. First, 1~2 mg of O-MMT was ground into a fine powder in an agate mortar to evenly mix it with dry potassium bromide powder. The mixture was then placed into a mold and pressed into a 150 μm thick film specimen using a tablet press. The infrared transmittance spectra were tested to identify the characteristic absorption peaks resulting from specific molecular group vibrations in O-MMT. The specified spectral range and scanning resolution were 400~4000 cm^−1^ and 0.8 cm^−1^, respectively.

An ultra-high-resolution cold field emission scanning electron microscope (SEM, SU8020, Hitachi Co., Tokyo, Japan) was utilized to analyze the dispersion and interface state of inorganic micron-nano fillers within an epoxy resin matrix. After being cooled with liquid nitrogen, the test composite material became brittle and broke easily. A brittle flake sample with a thickness of about 1 mm was taken. Finally, the obtained brittle flake samples were sprayed with a gold film on their cross-sections, followed by SEM tests.

Differential scanning calorimetry (DSC) was performed using a thermal analyzer (DSC-1, Mettler Toledo, Zurich, Switzerland) at a heating/cooling rate of 10 °C/min in a nitrogen atmosphere from 20 to 130 °C. The weight range for the test sample is specified as 5–10 mg.

### 2.4. Surface Electrical Conductivity Test

The three types of anti-corona paint samples were placed in the oven for a thermal oxygen aging test at 80 °C, 100 °C, and 120 °C, respectively. Subsequently, samples were taken out at time intervals of 168 h, 336 h, 504 h, and 672 h for testing.

To ensure the accuracy of the surface conductivity testing, the sample was placed in a dryer for 24 h prior to the test being conducted. The 1 min reading method was used to measure and record the conductance current of the sample at various voltages. A curve illustrating changes in conductivity based on filler type for the composite material was calculated and plotted.

The relationship between the electrical current *I* and the voltage *U* measured in the specimen is supposed to satisfy the following empirical equation:

(1) $$I=AU^{\alpha }$$

where *A* is a coefficient for describing the current–voltage relationship. Thus, the surface conductivity is calculated as follows:

(2) $$\sigma =\frac{K}{E}=\frac{I}{\pi D}\frac{d}{U}$$

where *σ* represents the surface resistivity, *d* indicates the distance between the electrodes, *D* denotes the diameter of the electrodes, and *E* signifies the testing electrical field strength. Taking *K* = *I*/*l*, *σ* = *K*/*E*, and *E* = *U*/*d* (*l* symbolizes the length of the electrodes) into Formulas (1) and (2), the following equation can be obtained:

(3) $$\sigma =BE^{\alpha -1}$$

The logarithm of Equation (3) is derived as lg*σ* = lg*B* + *β*lg*E*, where *β* is defined as the nonlinear coefficient and *B* is calculated as *B* = *Ad*^α^*l*^−1^. Eventually, the rate of surface conductivity variation could be defined as *r* = (*σ*_y_ − *σ*_x_)/*σ*_x_ where *σ*_y_ and *σ*_x_ denote the surface conductivities with and without aging, respectively. 

## 3. Results and Discussion

### 3.1. Material Characterization

#### 3.1.1. Infrared Spectrum Analysis

**Figure 5:**
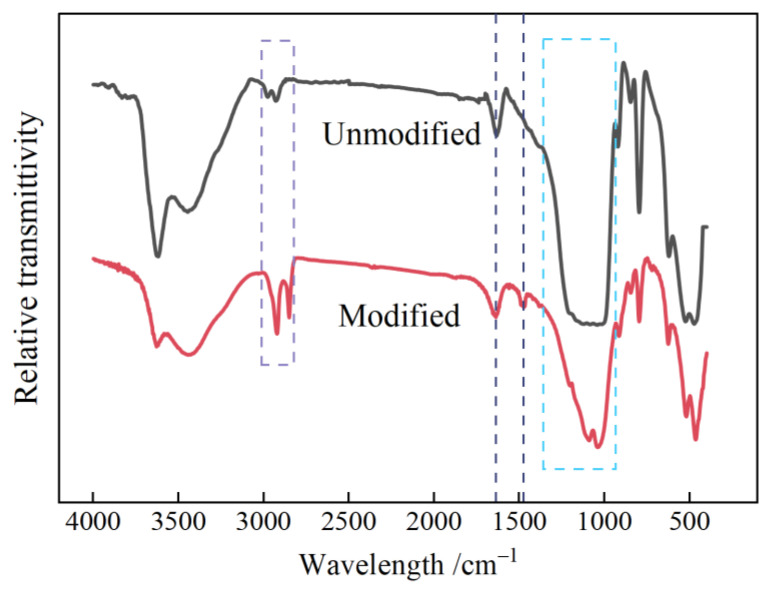
FTIR spectra of nano-scaled MMT before (unmodified) and after (modified) organic surface modification.

As shown in Figure 5, the FTIR spectra of MMT before and after undergoing organic surface modification reveal vibrational peaks at 2960~2850 cm^−^^1^, which correspond to long-chain methylene groups and indicate the presence of long-chain quaternary ammonium salt in the MMT layers. The flexural vibration peaks and C-N stretching vibration peaks of methyl methylene intercalation agents appeared at 1500–1450 cm^−^^1^ and 1290–1070 cm^−^^1^, respectively. This is because the N-O and C-N stretching peaks at 1030~1000 cm^−^^1^ and 1290~1070 cm^−^^1^, respectively, overlap with the Si-O stretching vibration absorption peaks of the MMT crystals at 1250–930 cm^−^^1^. The wavelength is more complex in the range of 950~690 cm^−^^1^, exhibiting multiple bending vibration peaks, such as AlAlOH bending, AlMgOH bending, the Platy form of tridymite, and Quartz. Al-O stretching and Si-O bending of montmorillonite appear at 540~450 cm^−^^1^. The presence of the N-O stretching vibration peak suggests that the introduction of a long-chain quaternary ammonium salt resulted in a stable bond formation with MMT lamellae. This enables the intercalation agent to remain stably incorporated within the lamellar structure of MMT, while also causing the expansion of the MMT lamellae. The vibrational absorption peaks associated with Si-O and Al-O bonds in MMT crystals were observed both before and after modification, indicating that the original lamellar arrangement of the MMT lattice remained intact following organic surface modification treatments. These results demonstrate that organic treatments are successful to fulfill surface modification on nano-scaled MMT [34,35]. The spectrum showed the characteristic vibration of the groups contained in MMT, as shown in Table 2 for detail.

#### 3.1.2. SEM Micro-Structure Characterization

**Figure 6:**
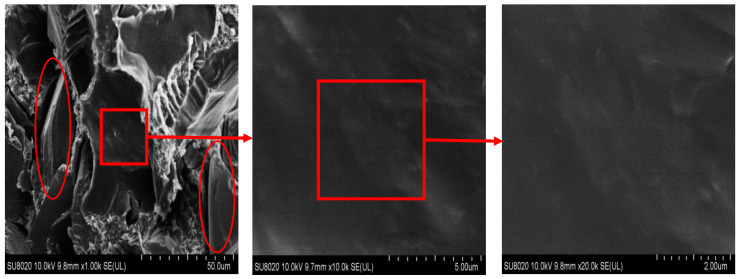
SEM image of SiC/EP micron composite. The magnification times from left to right are as follows: SEM images with magnification of 1000 times, 10,000 times, and 20,000 times.

**Figure 7:**
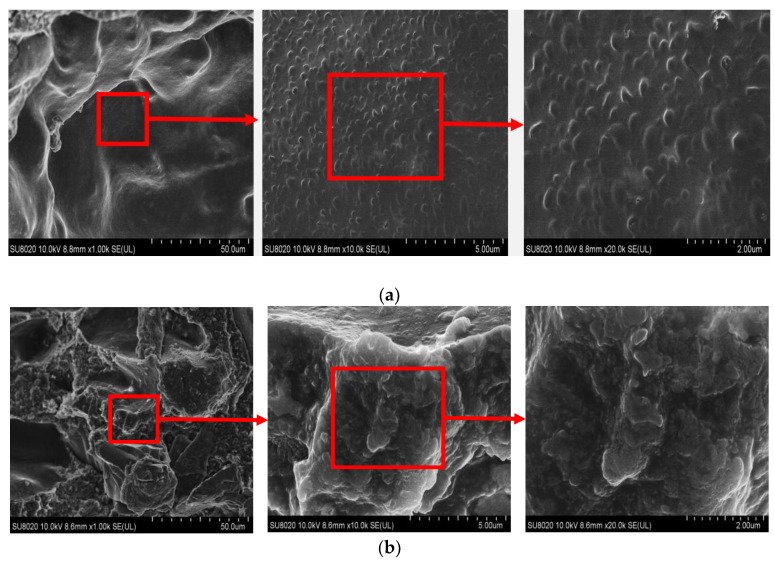
SEM image of anti-corona paint nanocomposites. The magnification times from left to right are as follows: SEM images with magnification of 1000 times, 10,000 times, and 20,000 times: (**a**) nano-SiC/EP; (**b**) O-MMT/SiC/EP.

The micromorphological image presented in Figure 6 demonstrates the uniform dispersion of micron-scale SiC particles within the epoxy resin matrix. Notably, the enclosed red oval highlights two micron silicon carbide particles, which exhibit limited interparticle contact despite a few instances of close proximity. Upon closer inspection using an enlarged low-acousto-light image, it becomes apparent that a smooth and flat matrix exists between these SiC particles. Moreover, the outlined red square area reveals no discernible presence of additional nanoparticles. SEM images magnified 1000, 10,000, and 20,000 times from left to right are shown in Figure 7. The images display the section of the nanoparticle located between two micron-scale silicon carbide particles that are evenly dispersed in the matrix, with no observed aggregation.

**Figure 8:**
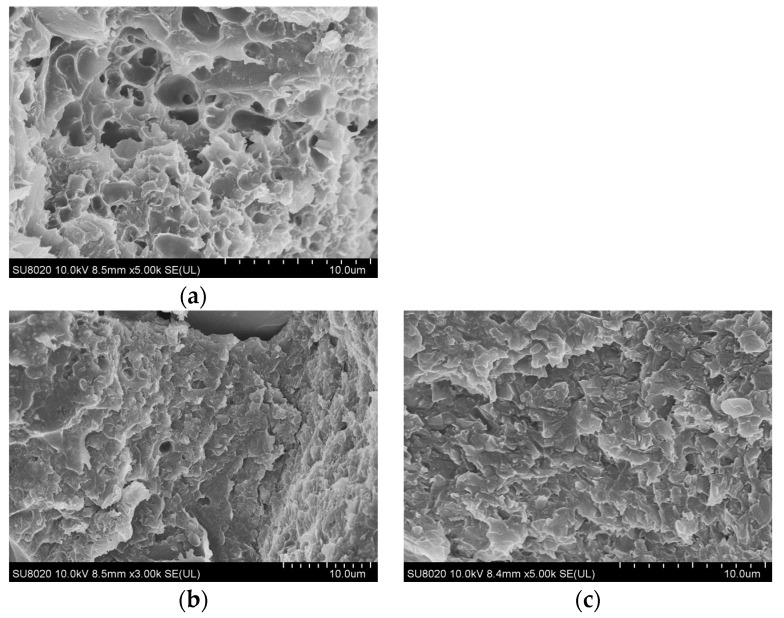
SEM images of thermo-oxygen-aged composites after aging at 120 °C temperature: (**a**) SiC/EP; (**b**) nano-SiC/EP; (**c**) O-MMT/SiC/EP.

As illustrated by the SEM images of the composites after thermal oxygen aging at 120 °C in Figure 8, the aged SiC/EP composite exhibits a porous and loosely structured appearance, characterized by the presence of air gaps. Conversely, the incorporation of nano-fillers effectively preserves the compactness of the composite matrix even after aging, resulting in reduced air gap formation compared to the SiC/EP matrix. Notably, the O-MMT/SiC/EP composite demonstrates the most densely packed matrix, with negligible air gap formation observed post aging.

The aging process of polymers in thermo-oxidative environments primarily occurs due to the facilitated ingress of oxygen into the material’s interior, induced by elevated temperatures. This phenomenon initiates aging at substantially lower temperatures than those required for thermal aging [36]. The lamellar morphology of organized O-MMT provides enhanced barrier effects compared to other nanofillers. This effect is twofold: firstly, the uniform dispersion of MMT flakes effectively impedes the spread of aging products to the exterior. Secondly, the lamellae act as a barrier, preventing oxygen from permeating into the polymer interior. The densely packed matrix impedes oxygen penetration, thereby partially mitigating the thermal oxygen aging process. Therefore, this feature contributes to superior thermal stability and resistance against heat and oxygen aging in the O-MMT/SiC/EP composite compared to other materials.

#### 3.1.3. DSC Analysis for Glass Transition Temperature

**Figure 9:**
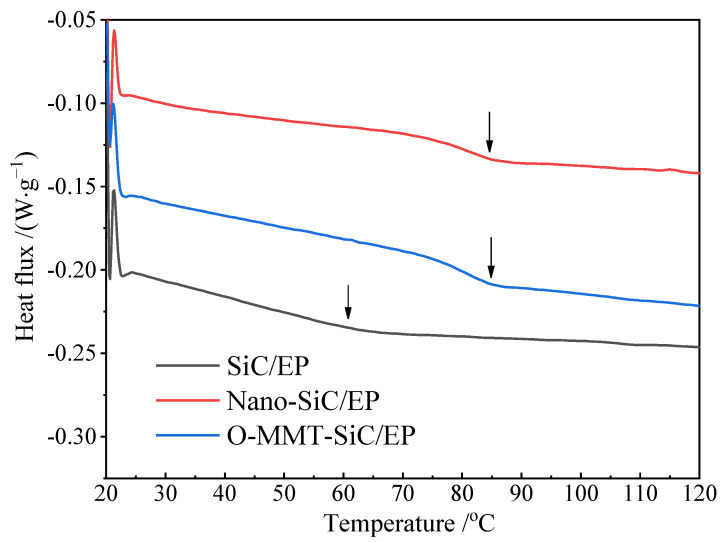
DSC temperature spectra of anti-corona paint composites.

The temperature at which the polymer transitions from a glassy state characterized by high strength to an elastic state with both high and low strength can serve as an indicator for assessing the material thermal resistance of materials. The glass transition temperature (T_g_) of composites serves as a crucial indicator for analyzing their thermal properties. As depicted in Figure 9 and summarized in Table 3, notable variations in T_g_ are observed across different specimens. In comparison to SiC/EP, both Nano-SiC/EP and O-MMT/SiC/EP exhibit elevated T_g_ values. Particularly, the T_g_ of O-MMT/SiC/EP rises to 85 °C.

This increase in T_g_ could be attributed to the binding effect exerted by micro- and nano-fillers on the molecular mobility of the polymer matrix [37]. According to the free volume theory of glass transition, the stronger the interaction between polymer molecules, the smaller the free volume becomes, resulting in a greater degree of constraint on the activity of polymer chains and an increase in T_g_. In addition, Strong interfacial interaction and large interfacial area play an important role in increasing the glass transition temperature [38,39,40]. 

### 3.2. Nonlinear Conductivity

**Figure 10:**
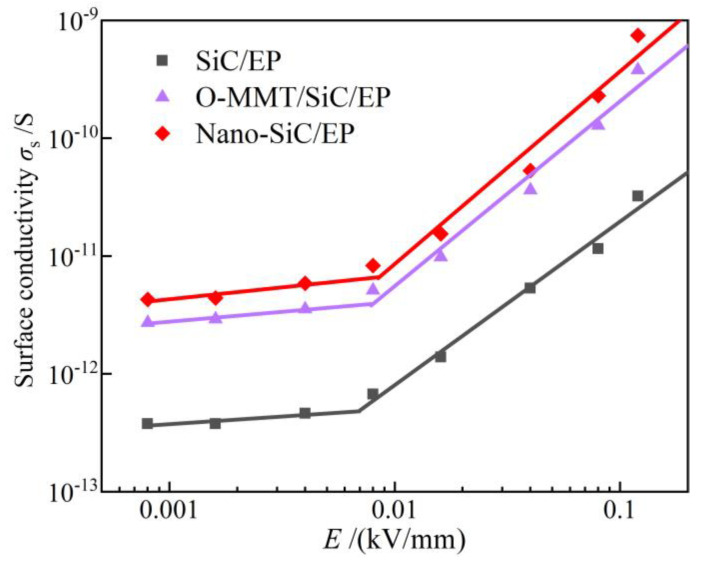
Conductivity vs. electric field strength of anti-corona paint composites.

The profiles of electrical conductivity versus electric field strength (σ~E characteristics) of three types of anti-corona composites are illustrated in Figure 10. It is worth noting that the inclusion of nano-SiC and O-MMT additives leads to a significant increase in conductivity compared to the benchmark SiC/EP composite. At a field strength of 0.08 kV/mm, the surface conductivities for Nano-SiC/EP and O-MMT/SiC/EP are 2.31 *×* 10^−10^ S and 1.29 × 10^−10^ S, respectively. Despite this enhancement, the threshold field intensity remains relatively unchanged, with all values hovering around 0.008 kV/mm. Notably, nano-SiC/EP exhibits a more pronounced variation in conductivity before and after reaching the threshold.

From a microscopic perspective, the conductivity of polymer dielectric composites is primarily influenced by carrier concentration and mobility. In the realm of solid dielectrics theory, conductance is typically categorized into ionic and electronic conductance. In weak fields, polymer carriers are primarily affected by intrinsic and weakly bound ionic conductance. The integration of nanofillers modifies the nonlinear conductivity properties of the composite dielectric. This phenomenon may stem from various factors, such as the presence of oxide layers on the surface of conductor or semiconductor filler particles, or interface states inducing the bending of surface charge and energy bands. Consequently, carrier concentration increases, leading to alterations in particle-to-particle contact and nonlinear carrier transport mechanisms, including thermal activation, jumping, tunneling, and barrier crossing [41,42].

The unique arrangement of the layer between grain boundaries contributes to the semiconductive properties of the composite dielectric. Trapped free electrons within this region create a depletion layer on the surface of each grain. When subjected to external electric fields, potential barriers at these boundaries shift, facilitating the directional movement of electrons through thermal motion and probabilistic mechanisms, thereby generating an electrical current.

### 3.3. Effect of Nanofiller Ageing on Nonlinear Conductivity

**Figure 11:**
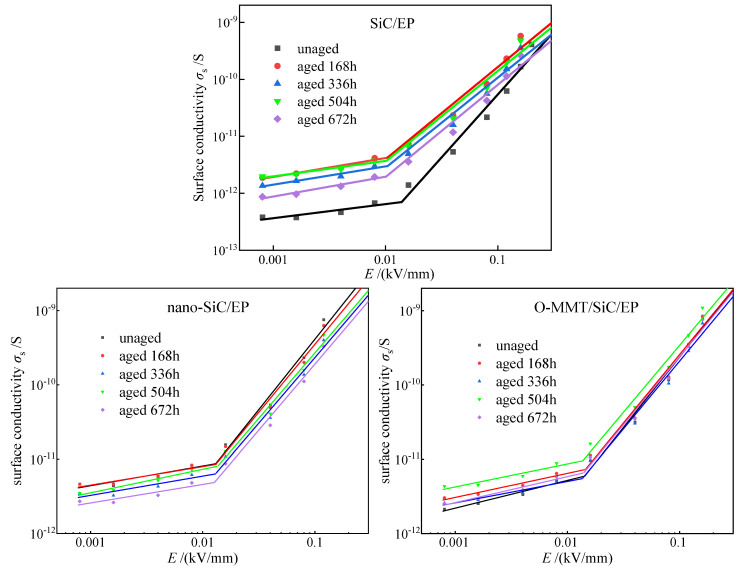
Electrical conductivity versus electric field strength of the SiC/EP, nano-SiC/EP, and O-MMT/SiC/EP composites before and after aging at 80 °C.

Figure 11 shows the electrical conductivity and electric field strength curves of the three composites before and after aging at 80 °C. It can be found from the figure that with the increase in aging time, the conductivity of each sample exhibited an overall increasing trend, and the conductivity also showed a certain fluctuation phenomenon. The conductivity mainly depends on the carrier concentration and mobility inside the material. Therefore, complex physical and chemical changes occur within each sample during the thermal aging process, resulting in alterations to either the carrier concentration or mobility inside the sample. This observed behavior can be attributed to several factors. Firstly, during the pre-aging phase, the degradation of residual curing agents due to heat results in the generation of free acid. The volatilization of this free acid from within the polymer matrix is hindered, thereby increasing conductivity by enhancing the number of free radicals within the material. As aging progresses, the epoxy resin’s main chain and the C-H bond on the alpha carbon atom near the benzene ring undergo degradation, resulting in the generation of additional free radicals. This degradation process, combined with physical changes such as thermal expansion and heat-induced material softening, leads to the formation of more noticeable defects on the surface of the material. The presence of these defects induces alterations in the electrical conductivity of the material. Secondly, thermal aging can induce potential damage to the molecular structure of the insulation material, subsequently disrupting its inherent ordered arrangement. This structural disruption may impede the pathway of electron transport, thereby reducing conductivity [43,44]. In addition, during the process of thermal aging, the insulation material may undergo oxidation with oxygen to generate oxides. These oxides could exhibit enhanced electrical resistivity, thereby leading to a reduction in overall conductivity [45].

**Figure 12:**
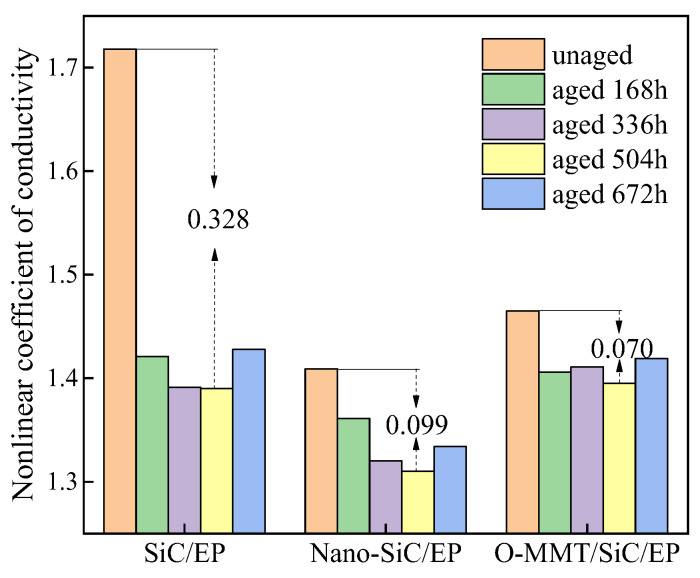
Nonlinear coefficients of the composites after thermo-oxidative aging at 80 °C under an electric field higher than the threshold field strength.

Figure 12 presents the nonlinear coefficients of the composites under the threshold field strength at the thermal oxygen aging temperature of 80 °C. As aging progresses, the magnitude of change in the nonlinear coefficients varies among composites with different fillers. Notably, the change in the nonlinear coefficients of MMT-filled composites is relatively small compared to those of other anti-corona paints. In particular, the O-MMT/SiC/EP composite exhibits a maximum nonlinear coefficient of 1.465 and a minimum of 1.395, with a difference of 0.07. This discrepancy may be attributed to the unique two-dimensional lamellar structure of O-MMT, which confers superior nonlinear conductivity properties compared to composites filled with zero-dimensional nanoparticles. As described in the previous SEM microscopic characterization, the layered morphology of the organized O-MMT provides a superior barrier effect compared to other nanofillers. The uniform dispersion of the MMT sheets effectively prevents the outward diffusion of aging products and inward penetration of oxygen into the polymer.

### 3.4. Effect of Nanofiller Aging Temperature on Nonlinear Conductivity

**Figure 13:**
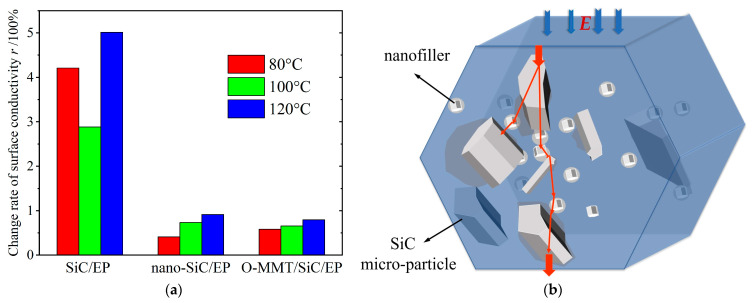
(**a**) Change rate of surface resistivity of the composites at different aging temperatures; (**b**) schematic surface conduction mechanism of nanodielectrics within multi-core model theory.

As shown by the variation in surface conductivity of the composites at different aging temperatures in Figure 13a, it is evident that the rate of change in surface conductivity is significant for SiC/EP at various aging temperatures. Conversely, anti-corona paints filled with nanofillers exhibit a markedly reduced rate of change in surface conductivity. This enhancement in thermal stability can be attributed to the incorporation of nanofillers, which effectively mitigate the deleterious effects of high aging temperatures on the epoxy resin matrix.

At elevated aging temperatures, the thermal expansion of the SiC/EP composite induces substantial contact failures among SiC particles within the EP matrix, impeding the formation of conductive pathways and resulting in a notable change in the conductivity of the SiC/EP composite. Conversely, according to the multi-core model theory, the decreased overlap interface of dielectric composites with nanoparticles post-aging is less pronounced owing to the large specific surface area of the nanofillers. This facilitates easy carrier transport across interfaces, thereby forming conductive pathways, as depicted in Figure 13b.

**Figure 14:**
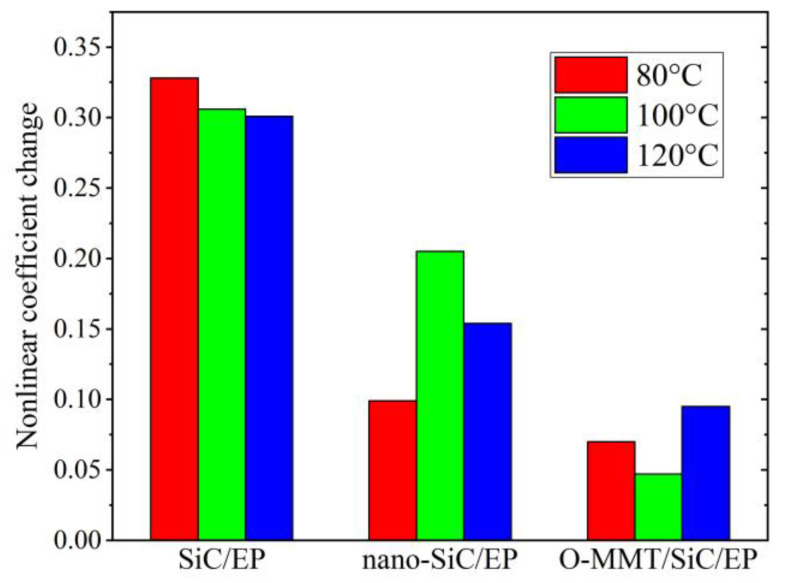
The variation magnitudes of nonlinear coefficients for the three anti-corona paint epoxy resin composites.

Moreover, the high surface energy of nanoparticles promotes adsorption on the molecular chains of epoxy resin, thereby reducing the polarization of macromolecular chains and diminishing potential barriers. Consequently, electrons are more prone to traversing lower potential barriers, leading to a minimal rate of change in the surface conductivity of epoxy composite media filled with nanoparticles at varying aging temperatures. Thus, the composite dielectric filled with nanofillers exhibited enhanced thermal stability, as depicted in Figure 14. Upon comparing the changes in nonlinear coefficients at different aging temperatures, it is evident that O-MMT/SiC/EP demonstrates the least variation, indicating superior thermal stability compared to other specimens.

## 4. Conclusions

In the present study, we investigate the impact of doping inorganic nanofillers into the SiC/EP matrix on the nonlinear conductivity and thermal stability of the epoxy composite dielectric, comparing the changes in the nonlinear coefficients of the three formulations. The glass transition temperatures of nano-SiC/EP and O-MMT/SiC/EP are notably enhanced, with the most significant increase observed up to 85.3 °C. Incorporating nanofillers such as nano-SiC and O-MMT leads to an increase in the surface conductivity of the anti-corona paint, while the threshold electric field strength exhibits minimal change. The rate of change in the surface conductivity of anti-corona paint filled with the nanofillers can be significantly reduced across different aging temperatures. Moreover, the magnitude of change in the nonlinear coefficients post-aging is minimal. Notably, the maximum nonlinear coefficient of O-MMT/SiC/EP reaches 1.465, while the minimum persists to 1.382, indicating the superior thermal stability of nonlinear conductance in O-MMT/SiC/EP. In summary, the addition of the proposed nanofillers can effectively improve the glass transition temperature and thermal stability in the nonlinear conductivity of the anti-corona paint. Specifically, O-MMT/SiC/EP exhibits the highest resistance of nonlinear conductance to glass transition and thermo-oxidative aging among the tested formulations.

## Figures and Tables

**Table 1:** Experimental materials.

Material Name	Notation	Product Manufacturer	Remarks
Silicon carbide /epoxy resin	SiC/EP	A large motor group Co., LTD, Chengdu, China	The content of micron silicon carbide in epoxy matrix is 72%. Average particle size of micron silicon carbide is 45 μm.
Nano silicon carbide	nano-SiC	Beijing Deco Island Gold Technology Co., Ltd., Beijing, China	Average particle size of 30 nm
Montmorillonite	MMT	Qinghe Chemical Factory, Zhangjiakou, Hebei, China	
Curing agent	593	Guangzhou Zhonggao Chemical Co., LTD, Guangzhou, China	Diethylenetriamine and butyl glycidyl ether. The relative density is 0.985. Soluble in ethanol, acetone, and other polar solvents. Reference dosage 18~28 copies. Curing condition room temperature/24 h.
Silane coupling agent	KH560	Saen Chemical Technology (Shanghai) Co., Ltd., Shanghai, China	
Octadecyl trimethyl ammonium chloride	S817662	Shanghai Macklin Biochemical Co., Ltd., Shanghai, China	

**Table 2:** FTIR band assignments for montmorillonite clay.

Maxima/cm^−1^	Tentative Assignment
3650–3400	–OH stretching, hydration
3000–2850	C–H asymmetric stretching of CTAB –CH_2_ stretching
1700–1600	–OH bending, hydration
1500–1450	–CH_2_—bending vibration
1290–1070	C-N stretching
1030–1000	N-O stretching
1250–930	Si–O stretching of montmorillonite
930–690	AlAlOH bending, AlFeOH bending, AlMgOH bending, Platy form of tridymite, Quartz
540–450	Al–O stretching and Si–O bending of montmorillonite

**Table 3:** Glass transition temperatures of anti-corona paint composites.

Composites	SiC/EP	Nano-SiC/EP	O-MMT/SiC/EP
T_g_*_/_*°C	60.0	84.5	85.3

## Data Availability

The datasets generated and/or analyzed during the current study are available from the corresponding author on reasonable request.
